# Antiulcerogenic Potential Activity of Free and Nanoencapsulated *Passiflora serratodigitata* L. Extracts

**DOI:** 10.1155/2014/434067

**Published:** 2014-07-13

**Authors:** Marc Strasser, Peky Noriega, Raimar Löbenberg, Nádia Bou-Chacra, Elfriede M. Bacchi

**Affiliations:** ^1^Department of Pharmacy, Faculty of Pharmaceutical Sciences, University of São Paulo, Professor Lineu Prestes 580 Avenue, Cidade Universitária, 05508-000 São Paulo, SP, Brazil; ^2^Faculty of Pharmacy and Pharmaceutical Sciences, University of Alberta, Avenue NW, Edmonton, AB, Canada T5M 3X3

## Abstract

This paper provides evidence that the leaves and stem of *Passiflora serratodigitata* L. dry crude extract (DCE), ethylacetate fraction (EAF), and residual water fraction show potential antiulcerogenic activity. Interestingly, the polymeric nanocapsule loaded with EAF had 10-fold more activity than the free EAF. Furthermore, the polymer nanoparticles provided homogeneous colloidal drug delivery systems and allowed overcoming challenges such as poor aqueous solubility as well as the physical-chemical instability of the organic extract, which presented 90% (w/w) of the flavonoid content. The entrapment efficiency of the total flavonoid was 90.6 ± 2.5% (w/v) for the DCE and 79.9 ± 2.7% (w/v) for the EAF. This study shows that nanoencapsulation improves both the physicochemical properties and the efficacy of the herbal formulations. Therefore, free and encapsulated extracts have the potential to be suitable drug design candidates for the therapeutic management of ulcer.

## 1. Introduction

The Passifloraceae family includes more than 600 species of tropical and subtropical origin; 90% of these species are native to the Americas and more than 200 species are native to Brazilian areas [1]. The antiulcer properties of genus* Passiflora* L. (Passifloraceae) has recently been investigated, suggesting the use of plants and their extracts as potential therapeutic remedies. Extracts from whole* P. foetida *plants showed antiulcer and antioxidant activities [2]. To the best of our knowledge, the antiulcerogenic activity of* P. serratodigitata *species has not yet been reported. Peptic ulcer disease (PUD) has a large impact on the worldwide health care system. Therefore, a cure for ulcers would result in large economic and medical savings [3]. PUD is most commonly associated with* Helicobacter pylori* infection and the use of acetylsalicylic acid (ASA) [4] and nonsteroidal anti-inflammatory drugs (NSAIDs) [5]. The management of* H. pylori* infection has improved radically in recent years; however, the prescription of ASA and NSAIDs has increased over the same period [6]. Moreover, the etiology of gastroduodenal ulcers is influenced by various additional aggressive and defensive factors such as dietary habits, stress, acid-pepsin secretion by the parietal cells, the mucosal barrier, mucus secretion, blood flow, cellular regeneration, and endogenous protective agents (prostaglandins and epidermal growth factors) [7]. In recent years, interest in alternative therapies has been growing, and particular interest in plant sources is developing in light of their potential lower side effects, availability, and cost-effectiveness. Plants are some of the most attractive sources of new drugs, and some have shown promise in the treatment of gastroduodenal ulcers with minimal side effects [3]. Herbal medicine is one of the oldest sciences throughout the world. However, the development of natural products is a challenging task due to the complexity of plant extracts. Formulators must overcome intrinsic challenges such as poor aqueous solubility and permeability as well as the physical-chemical instability of many herbal extracts. However, advancements in extraction technologies have helped to transform ancient formulas into modern therapeutic remedies [8]. Furthermore, modern drug delivery approaches can enable the development of a new generation of herbal medicines. This is a rapidly developing area in biomedical research that promises advances and breakthroughs in therapeutic outcomes [9]. The application of nanoscale delivery systems to herbal remedies may provide remarkable advantages over conventional formulations by improving component solubility, enhancing bioavailability, reducing dose, achieving constant therapeutic levels over an extended time, enhancing stability, and protecting the compounds from physical and chemical degradation [10–12]. Several studies have confirmed these advantages. For example, thymoquinone, a phytochemical compound extracted from* Nigella sativa*, has anti-inflammatory, antioxidant, and anticancer effects. Moreover, thymoquinone-loaded lipid nanocarriers showed gastroprotective effects [13]. Curcumin, a natural but poorly soluble polyphenol isolated from turmeric (*Curcuma longa*), also had antioxidant and anticancer activities. A polymeric curcumin nanoparticle system allowed researchers to improve its solubility and enhance its dissolution mechanism [14]. Recently, a nanoencapsulated form of* Phytolacca decandra* ethanolic extract showed better* in vivo* chemopreventive action against lung cancer than its free extract [15]. Furthermore,* Emblica officinalis* extract encapsulated in polymeric nanoparticles showed a linear release of the active ingredient. This plant, a commonly available fruit plant in the tropical and subtropical regions of India, has several medicinal properties [16]. Additionally, the polymeric nanoparticles containing an ether extract from the root of* Clerodendrum infortunatum* L. showed potential use in the treatment of hypercholesterolemia by passively targeting nanoparticles to the liver [17]. Thus, nanoscale herbal drug delivery systems may enhance the activity of drugs and overcome other issues related to conventional medicines [18]. The goal of this study was to evaluate the potential antiulcerogenic activity of free and nanoencapsulated extracts from the leaves and stems of* Passiflora serratodigitata* L. against the ethanol/acid-induced gastric ulcer model in rats.

## 2. Materials and Methods

### 2.1. Materials

The leaves and stems of* Passiflora serratodigitata *L. (Passifloraceae) were collected and identified by Luis Carlos Bernacci from the Agronomic Institute of Campinas, São Paulo, Brazil. A voucher specimen was deposited at the herbarium under sample number 37,969. The solution of standard quercetin, the sorbitan monostearate (span 60), and the poly(epsilon-caprolactone) (PCL) were purchased from Sigma-Aldrich. Capric/caprylic triglyceride (Miglyol 182) was bought from Quantiq (São Paulo, Brazil). Polysorbate 80 was purchased from Oxiteno (São Paulo, Brazil). Ethylacetate, acetone, ethanol 60% (v/v), and 0.3 M hydrochloric acid were of analytical grade and were used as supplied. Lansoprazole was used as a control treatment.

### 2.2. Preparation of the 60% (v/v) Hydroalcoholic Extract

The leaves and stem of* Passiflora serratodigitata *L. were dried in an oven with circulating air at 40°C for a week (Fabbe, Brazil) and then milled in a knife (Arthur Thomas Co, USA) and hammer mill (Metalurgica Roma, Brazil). The hydroalcoholic extract was obtained by percolation as described by the Brazilian Pharmacopoeia [19], briefly, the dried and milled leaves and stem were moistened and transferred to a percolator with an appropriate amount of ethanol 60% (v/v). The mixture was allowed to macerate in the closed percolator for 24 h. The extract was subsequently obtained by percolation. The obtained extract was concentrated using a rotary evaporator at reduced pressure (model R-215, Buchi, Switzerland), and the remaining aqueous solution was lyophilized (LK4 Edwards, USA), resulting in a dry crude extract (DCE).

### 2.3. Preparation of the Organic and Residual Aqueous Fraction Extracts

**Figure 1:**
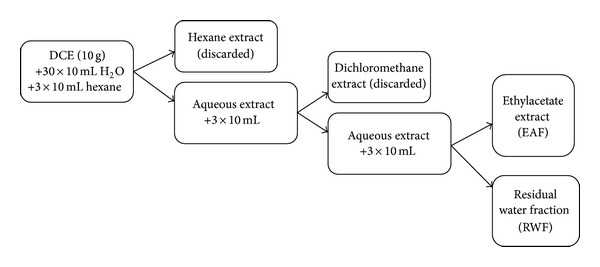
Schematic preparation of the ethylacetate extract (EAF) and the residual water fraction (RWF). DCE: dry crude extract.

The DCE (10 g) was dispersed in 30 mL of water to obtain a suspension. Total of 3 portions of 10 mL of hexane were added to the obtained suspension. The hexane fraction was discarded, and the remaining aqueous extract was treated with 3 portions of 10 mL of dichloromethane. The dichloromethane was again discarded, and the same procedure was applied to the remaining aqueous extract using ethylacetate. The last remaining aqueous extract was called the residual water fraction (RWF). The ethylacetate fraction (EAF) was concentrated and dried using a rotary evaporator at reduced pressure (model R-215, Buchi, Switzerland), and the RWF was lyophilized (lLK4, Edwards, USA) (Figure 1).

### 2.4. Determination of Total Flavonoid Content

A spectrophotometric assay to determine flavonoid content was based on the formation of an aluminum chloride complex at 415 nm, and quercetin was used as the marker compound [20]. A 10 mg/mL standard solution of quercetin was prepared using 50% (v/v) methanol to obtain a 5-point standard curve (50, 100, 150, 200, and 250 *μ*g/mL; *n* = 3). The assay was checked for potential interference from the excipients used for the encapsulation. The linearity of the calibration process was evaluated by means of the lack-of-fit test, Durbin-Watson statistic (DW), and the coefficient correlation (*r*). Furthermore, the residual plots were evaluated to validate the selected regression model.

### 2.5. Nanoencapsulation

Nanoencapsulated extracts were obtained by a solvent displacement technique [15]. Poly(epsilon-caprolactone) (PCL) (0.010 g) was dissolved in acetone, a water-miscible solvent of intermediate polarity, by stirring (200 rpm) for 40 minutes at 40°C (organic phase). A total of 0.333 g of capric/caprylic triglyceride and a total of 0.077 g of sorbitan monostearate (span 60) were added to the organic phase. The DCE and EAF extracts were added after the polymer dissolved and allowed to stir for 10 minutes in the organic phase (200 mg or 20 mg/mL for the DCE, and 50 mg or 5 mg/mL for the EAF). The organic phase containing the DCE was filtered to remove the insoluble particles (Non-Sterile 25 mm Millex-LS, pore size: 5 *μ*m, syringe filter unit, Millipore). The addition of EAF to the organic phase resulted in a clear solution. The organic phase was injected into a stirred aqueous solution (amount of water: 53 mL) containing 0.070 g of polysorbate 80. Polymer deposition at the interface of the solvents leaded to the instantaneous formation of a colloidal suspension. The organic solvent was removed using reduced pressure in a rotary evaporator (model R-215, Buchi, Switzerland), and the final aqueous volume was adjusted to 10 mL with water. Only lipophilic compounds can be trapped using this method (lipid core nanoparticles). Thus, the RWF was not nanoencapsulated due to its solubility characteristics (i.e., it is water soluble). The encapsulated DCE and EAF were referred to as the NDC extract and NEAF, respectively.

### 2.6. Characterization of the Encapsulated Extracts

The average particle size and the polydispersity index (PDI) were measured by DLS (dynamic light scattering) using a Zetasizer ZN particle size analyzer (Malvern Instruments, Southborough, UK) equipped with vertically polarized light supplied by an argon-ion laser. The Zeta potential was also determined by DLS (Zetasizer ZN particle size analyzer, Malvern Instruments, Southborough, UK). All the measurements were determined from the mean values of three experiments. The entrapment efficiency (EE) was measured by determining the total and free flavonoid concentration. To determine the amount of free flavonoid, 1 mL of the suspension was transferred to an ultrafilter (Microcon Ultracel YM-10, Millipore) and centrifuged (VWR, Galaxy 16D) at 8,000 rpm for 30 minutes. The filtrate was used to determine the free flavonoid in the extracts. To determine the amount of total flavonoid, the formulations were dissolved using acetonitrile to extract the flavonoid content.

### 2.7. Ethanol/Acid-Induced Gastric Ulcer Model in Rats

The study of acute gastric injury in rats was approved by the Research Animal Ethics Committee, Faculty of Pharmaceutical Sciences, University of São Paulo (approval identification: CEEA no. 234). This protocol was previously described by Patil et al. [21]. Briefly, 7-week-old Wistar rats (weighing between 150 and 180 g) were fasted for twelve hours with access to water* ad libitum *until the start of the experiment. The animals were exposed to the following preparations by gavage: the DCE at concentrations of 100, 200, and 400 mg/kg bodyweight, or the EAF at a concentration of 50 mg/kg bodyweight. Purified water (1 mL/100 g bodyweight) and 30 mg/kg bodyweight lansoprazole were used as a negative control and a positive control, respectively. Thirty minutes after administration, the animals received 1 mL/100 g bodyweight of a solution of 0.3 M hydrochloric acid in 60% (v/v) ethanol as an ulcerative agent. One hour later, the animals were euthanized in a CO_2_ cage, and their stomachs were removed surgically. The stomachs were scanned, and representative pictures were recorded and analyzed using Image Pro Plus (Media Cybernetics, USA) software. The analysis was performed using the following parameters: ulcerated lesion total area (ULTA) in mm^2^, calculated by the sum of the areas of individual lesions, and ulcerated lesion relative area (URLA), expressed as the percentage of ulcerative area (p) in relation to the total area of the stomach and the percentage of protected tissue, which refers to the percentage of noninjured area in comparison with the negative control. These area percentages were transformed using the arcsine formula (provided by the software) for appropriate statistical analysis.

### 2.8. Statistical Analysis

The ulcer lesion areas are represented as the mean ± the standard error of the mean, and the level of ulcer protection is presented as a percentage inhibition. The significance of the differences in the mean ulcer indices between the extracts and negative and positive controls was calculated at a 95% confidence interval using ANOVA followed by Tukey's range test.

## 3. Results and Discussion

### 3.1. Determination of Total Flavonoid Content

**Figure 2:**
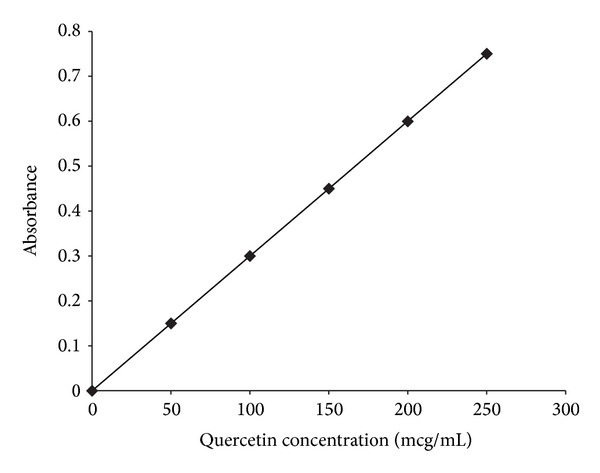
Standard calibration curve of quercetin.

The linearity of the calibration curve of quercetin had a correlation coefficient of 0.995 (Figure 2) and a Durbin-Watson (DW) statistic equal to 2.13, and the lack-of-fit test showed *P* value equal to 0.689 (*α* = 0.05). The residual plot analysis showed constant variance, independence of variables, and normality of the distribution. In addition, the observed residuals were randomly distributed around zero (data not shown). The evaluation of these statistics and the residual plots showed the goodness of fit of the model. The total flavonoid content of the* Passiflora serratodigitata* stem and leaf dry crude (DCE) and ethylacetate fraction extracts (EAF) was 116.99 ± 6.14 and 105.50 ± 4.59 *μ*g/mg, respectively (Table 1).

### 3.2. Characterization of the Encapsulated Extracts

In this study, biodegradable nanoparticles were prepared using poly(epsilon-caprolactone). The NDC and NEAF* Passiflora* extracts showed mean particle diameters equal to 379.2 ± 16.4 and 383.8 ± 18.2 nm, and the zeta potentials were −20.2 ± 1.8 and −27.3 ± 1.1, respectively (Table 2). The entrapment efficiency of the total flavonoid was 90.6 ± 2.5% (w/v) for the DCE and 79.9 ± 2.7% (w/v) for the EAF extract (Table 1). Polymeric nanoparticles have been extensively used as drug delivery systems for a variety of plant extracts, providing similar results. Several studies [15–17, 22, 23], including the present one, revealed that the nanoencapsulation method led to a more homogeneous colloidal drug delivery system.

### 3.3. Antiulcerogenic Activity

**Figure 3:**
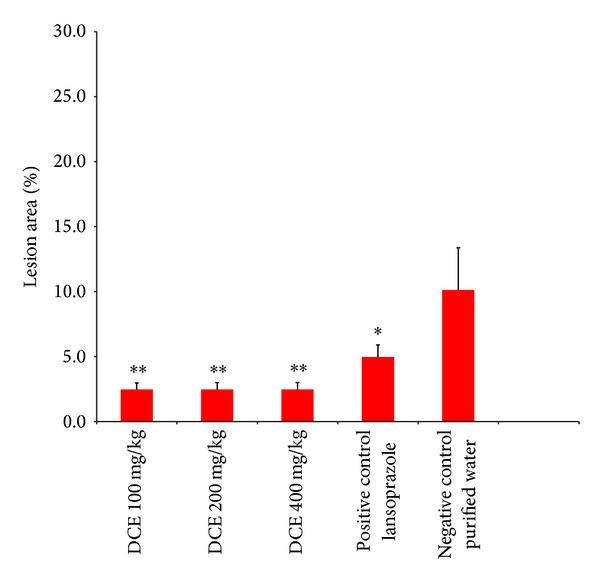
The antiulcer activity of the dry crude extract (DCE) of* Passiflora serratodigitata *L. *:*P* < 0.05, **:*P* < 0.01 compared to the negative control (purified water) (*n* = 7 for each group). The dose of lansoprazole used in this experiment was 30 mg/kg.

Most experiments that study peptic ulcers are carried out in rodents. The ethanol-induced ulcer model is useful for studying the efficacy of potential drugs or testing agents that have cytoprotective and/or antioxidant activities [7]. The antiulcerogenic activity studies showed that the DCE protected the stomach against necrotic damage due to HCl/ethanol; the effect of the DCE was more pronounced than that of lansoprazole (30 mg/Kg), a proton-pump inhibitor (PPI), which prevents the stomach from producing gastric acid. The DCE significantly (*P* value < 0.05, *α* = 0.05) reduced the relative lesion area at 100 mg/Kg, 200 mg/Kg, and 400 mg/Kg to 2.5% (Figure 3). Given the tested concentrations, it was not possible to observe a dose-response relationship (100, 200, and 400 mg/Kg) for the antiulcerogenic activity of DCE (Figure 3), because the maximum protective effect was achieved at 100 mg/Kg. Similar antiulcerogenic activity of an ethanolic extract of* Passiflora foetida* L. whole plant was recently reported by Sathish et al. [2]. In this rat study, the antiulcer activity was investigated using an ethanol and aspirin-induced gastric ulcer model at doses of 100 and 200 mg/Kg.

**Figure 4:**
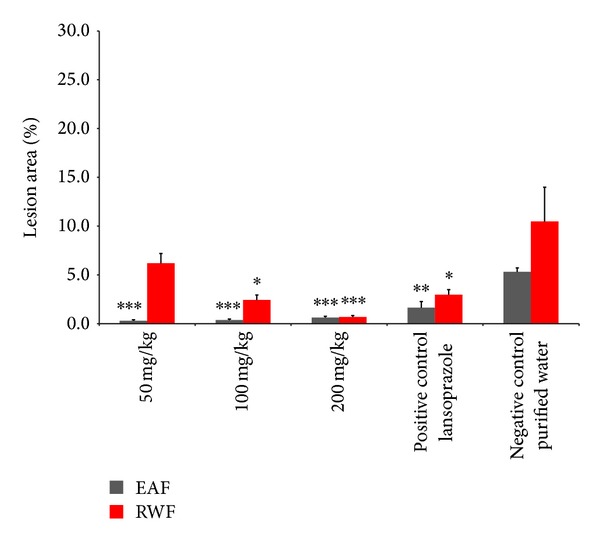
Comparison between the antiulcer activities of the ethylacetate fraction (EAF) (grey bars) and the residual water fraction (RWF) (red bars) of* Passiflora serratodigitata* L. *:*P* < 0.05, **:*P* < 0.01; ***:*P* < 0.001 compared to the negative control (purified water for both assays) (*n* = 7, for each group for both assays). A dose of 30 mg/kg lansoprazole was used for both assays.

The RWF extract of* Passiflora serratodigitata* showed a dose-dependent antiulcer activity. A dose of 50 mg/kg RWF was less effective in protecting the gastric wall than the 100 and 200 mg/kg doses (Figure 4). In contrast to the RWF, the EAF showed no dose response; this is probably due to the effect observed for the DCE: the lowest tested concentration (50 mg/Kg) showed a maximum protective effect. However, the relative protection of the EAF was more pronounced compared to the DCE, most likely as a result of the higher purity of this extract compared with the crude one. There was no statistically significant difference in the antiulcerogenic activity of the RWF and the EAF extracts 200 mg/Kg (Figure 4). These results indicated that the flavonoid C-glycoside (hydrophilic flavonoid) and the other hydrophilic components detected by the phytochemical screening (alkaloid and tannin (data not shown)) also play a role in the observed antiulcerogenic activity.

**Figure 5:**
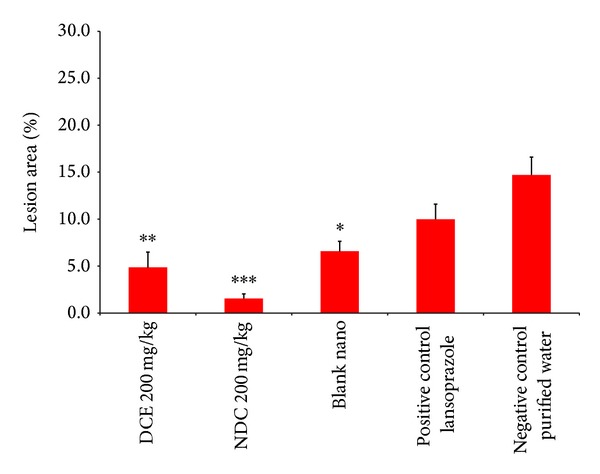
The antiulcer activity of the dry crude extract (DCE) and the nanostructured dry crude (NDC) extract of* Passiflora serratodigitata* L. *:*P* < 0.05, **:*P* < 0.01 compared with negative control (purified water) and blank nanoparticles (*n* = 7 for each group). A 30 mg/Kg dose of lansoprazole was used as a positive control.

The NDC* Passiflora* extract had approximately 4 times more protection than the DCE at the same concentration (200 mg/Kg). Nanoencapsulation improved the bioactivity of the extract (Figure 5). Similarly, Bisht et al. [23] reported an improved bioactivity of curcumin using polymeric-based nanoparticles. Moreover, the authors reported that nanoscale formulated curcumin provided better dispersibility of the active ingredient. The present study revealed similar results. The physical properties of the nanoencapsulated* Passiflora* extracts improved the flowability, and the formulations could be easily dispersed in water, while the EAF extract was more difficult to disperse.

The blank nanocapsules also showed some stomach protection (Figure 5), most likely due to the formation of a physical barrier film protecting the stomach against the ulcerogenic agents (HCl/ethanol). To the best of our knowledge, the protective effect of empty nanoparticles has not been reported before. Thus, we will make an effort to elucidate this protective mechanism in the near future.

**Figure 6:**
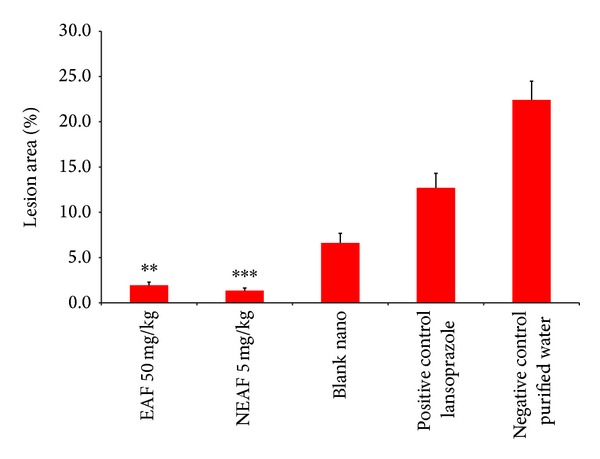
Comparison of the antiulcer activities of the ethylacetate fraction (EAF) of* Passiflora serratodigitata* L. (50 mg/kg) and the nanostructured EAF (NEAF) (dose 5 mg/kg), **:*P* < 0.01, ***:*P* < 0.001 compared to the negative control (purified water) and blank nanoparticles. Lansoprazole (30 mg/Kg) was used as a positive control (*n* = 7 for each group).

The NEAF* Passiflora* extract required 10 times less extract (5 mg/Kg) for the same activity than the EAF extract (Figure 6). For the DCE, the encapsulated form (NDC) had 4-fold more antiulcerogenic activity than its free form (Figure 5). The NEAF was more potent compared to the NDC extract, most likely due to its higher purity.

Therefore, the NEAF and NDC extracts further improved their efficacy due to the nanosize formulation. This may be because these formulations rendered the extracts more water soluble and improved dispersion in the stomach wall. Nevertheless, the antiulcerogenic activity of any individual phytocomponent of the stem and leaf extracts has not yet been investigated and may shape the foundation for future studies. Thus, until such studies are conducted, the significant antiulcerogenic activity found in this work could currently be attributed to the total flavonoid content in the extracts.

## 4. Conclusion

Three* Passiflora serratodigitata* L. extracts (crude (DCE), ethylacetate (EAF), and residual water fraction (RWF)) showed antiulcerogenic activity. The nanoencapsulated extracts (NDC and NEAF) had greater antiulcerogenic activity than their conventional extract forms. This study shows that nanoencapsulation improves both the physicochemical properties and the efficacy of the herbal formulations. Therefore, free and encapsulated extracts have the potential to be suitable drug design candidates for the therapeutic management of ulcers.

## Figures and Tables

**Table 1:** Total flavonoid content in the *Passiflora serratodigitata* stem and leaf dry crude extract (DCE), ethylacetate fraction (EAF) extract, and the entrapment efficiency (EE) (*n* = 6).

Extracts	Total flavonoid (*µ*g/mg extract)	EE % (w/v)	Entrapped total flavonoid mg/mL
DCE	116.99 ± 6.14	90.6 ± 2.5	2.120∗
EAF	105.50 ± 4.59	79.9 ± 2.7	0.528∗∗

(∗) in 200 mg; (∗∗) in 50 mg.

**Table 2:** Physicochemical characterization of nanostructured *Passiflora serratodigitata *leaf and stem extracts.

Extract	Mean particle size (nm)	Polydispersity index	Zeta potential (mV)
NDC	379.2 ± 16.4	0.253 ± 0.021	−20.2 ± 1.8
NEAF	383.8 ± 18.2	0.353 ± 0.034	−27.3 ± 1.1
Blank	245.1 ± 10.2	0.172 ± 0.009	−53.6 ± 2.9

NDC: nanostructured dry crude extract; NEAF: nanostructured ethylacetate fraction.
